# Efficient and Stable Planar n–i–p Sb_2_Se_3_ Solar Cells Enabled by Oriented 1D Trigonal Selenium Structures

**DOI:** 10.1002/advs.202001013

**Published:** 2020-06-30

**Authors:** Kai Shen, Yu Zhang, Xiaoqing Wang, Chizhu Ou, Fei Guo, Hongbing Zhu, Cong Liu, Yanyan Gao, Ruud E. I. Schropp, Zhiqiang Li, Xianhu Liu, Yaohua Mai

**Affiliations:** ^1^ Institute of New Energy Technology College of Information Science and Technology Jinan University Guangzhou 510632 China; ^2^ Institute of Photovoltaics Hebei University Baoding 071002 China; ^3^ National Engineering Research Center for Advanced Polymer Processing Technology Zhengzhou University Zhengzhou 450002 China

**Keywords:** high efficiency, n–i–p structure, orientation, Sb_2_Se_3_ solar cells, trigonal selenium

## Abstract

Environmentally benign and potentially cost‐effective Sb_2_Se_3_ solar cells have drawn much attention by continuously achieving new efficiency records. This article reports a compatible strategy to enhance the efficiency of planar n–i–p Sb_2_Se_3_ solar cells through Sb_2_Se_3_ surface modification and an architecture with oriented 1D van der Waals material, trigonal selenium (t‐Se). A seed layer assisted successive close spaced sublimation (CSS) is developed to fabricate highly crystalline Sb_2_Se_3_ absorbers. It is found that the Sb_2_Se_3_ absorber exhibits a Se‐deficient surface and negative surface band bending. Reactive Se is innovatively introduced to compensate the surface Se deficiency and form an (101) oriented 1D t‐Se interlayer. The p‐type t‐Se layer promotes a favored band alignment and band bending at the Sb_2_Se_3_/t‐Se interface, and functionally works as a surface passivation and hole transport material, which significantly suppresses interface recombination and enhances carrier extraction efficiency. An efficiency of 7.45% is obtained in a planar Sb_2_Se_3_ solar cell in superstrate n–i–p configuration, which is the highest efficiency for planar Sb_2_Se_3_ solar cells prepared by CSS. The all‐inorganic Sb_2_Se_3_ solar cell with t‐Se shows superb stability, retaining ≈98% of the initial efficiency after 40 days storage in open air without encapsulation.

## Graphical abstract

This work reports a compatible strategy to enhance the efficiency of planar n–i–p Sb_2_Se_3_ solar cells through Sb_2_Se_3_ surface modification and an architecture with oriented 1D van der Waals material, trigonal selenium (t‐Se). The p‐type t‐Se layer functionally works as a surface passivation and hole transport material. The all‐inorganic device structure enables high efficiency and superb stability.

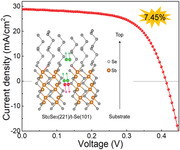

## 1 Introduction

Quasi‐1D(Q1D) V–VI antimony chalcogenide‐based solar cells have drawn intensive attention as a result of the meteoric rise in power conversion efficiencies over the past few years.^[^ ^1^, ^2^, ^3^, ^4^, ^5^, ^6^, ^7^, ^8^, ^9^, ^10^, ^11^, ^12^, ^13^, ^14^ ^]^ In particular, antimony selenide (Sb_2_Se_3_) has recently emerged as a highly attractive light‐harvesting material arising from its extraordinary properties such as high absorption coefficient (>10^5^ cm^−1^), suitable optical bandgap (1.1–1.3 eV), good carrier mobility (≈10 cm^2^ V^−1^ s^−1^), and simple single phase structure.^[^ ^1^, ^2^, ^3^, ^4^, ^5^, ^14^, ^15^ ^]^ The earth‐abundant low‐toxic materials, together with scalable and cost‐effective fabrication processes promise Sb_2_Se_3_ solar cells a competitive technology for high‐efficiency, low‐cost photovoltaics. The unique Q1D structure of Sb_2_Se_3_ enables intrinsically benign grain boundaries if the crystals are properly aligned.^[^ ^2^ ^]^ To date, the highest reported power conversion efficiencies of both superstrate‐planar and substrate‐nanorod type Sb_2_Se_3_ solar cells are 7.6% and 9.2%, respectively.^[^ ^4^, ^5^ ^]^

Nevertheless, the low carrier density of Sb_2_Se_3_ and high interface recombination loss represent the two most urgent obstacles to further improvement of the device efficiency.^[^ ^16^, ^17^ ^]^ The intrinsic defects in the low‐symmetry Sb_2_Se_3_ semiconductors are complicated and limit their p‐type doping level.^[^ ^18^ ^]^ To obtain high‐quality Sb_2_Se_3_ absorbers, several approaches have been developed, such as rapid thermal evaporation,^[^ ^1^, ^2^ ^]^ close spaced sublimation (CSS),^[^ ^5^, ^19^, ^20^ ^]^ vapor transport deposition.^[^ ^3^, ^4^, ^21^, ^22^, ^23^ ^]^ Early work mainly focused on improving the quality of the absorber (orientation control, deep defect passivation) and on engineering junction interfaces.^[^ ^1^, ^2^, ^3^, ^4^ ^]^ To further improve device performance, increasing attention has been paid to the trapping and carrier transport at the Sb_2_Se_3_ rear surface and related interface. Tang and co‐workers reported that, due to the electrical loss induced by rear surface/interface defects and the presence of a contact barrier, the additional deficit of open‐circuit voltage of Sb_2_Se_3_ solar cells can exceed 0.2 V.^[^ ^16^ ^]^ Nevertheless, little attention has been paid to the properties of the Sb_2_Se_3_ rear surface, despite their role in limiting device performance. In this regard, effective surface treatment or passivation methods are yet to be developed to further advance the device efficiency.

In addition to the lack of well‐established surface modification methods, most of the existing superstrate Sb_2_Se_3_ cell structures do not have a wide‐bandgap barrier/passivating layer at the absorber interface to provide carrier confinement and reduce interface recombination velocity. Furthermore, considering the quasi‐intrinsic nature of the Sb_2_Se_3_ absorber layer, incorporation of a p‐type interfacial layer to construct n–i–p configuration is a feasible way to overcome the bottleneck of the low doping concentration (10^13^ cm^−3^, as i‐layer) of the absorber layer and improve the carrier extraction efficiency.^[^ ^17^, ^24^ ^]^ Exploration of efficient charge‐extraction material or hole‐transporting material (HTM) is the key to construct n–i–p Sb_2_Se_3_ solar cells. Recently, organic materials (Spiro‐OMeTAD, CZ‐TA, PCDTBT) and inorganic materials (PbS quantum dot, CuSCN, NiO) have been investigated as HTMs to improve device performance (Figure S1, Supporting Information).^[^ ^16^, ^17^, ^20^, ^22^, ^24^, ^25^ ^]^ Especially, PbS quantum dots have been successfully used to prepare record‐efficiency (7.62%) Sb_2_Se_3_ solar cells.^[^ ^4^ ^]^ However, the toxicity of Pb, the high‐mobility Cu impurity and the instability of organic materials limit their practical applications. An ideal interfacial structure should feature compatible material composition, matched energy band level, low interface defect density, good conformal coverage, and low‐contact barrier. Meeting all these requirements is not easy but essential to realize efficient Sb_2_Se_3_ devices.

In this work, we present a strategy that uses oriented trigonal selenium (t‐Se) to modify the Sb_2_Se_3_ surface and construct n–i–p Sb_2_Se_3_ solar cells. Different from the ring structure of selenium clusters, trigonal selenium is a 1D van der Waals solid with helical atomic chains.^[^ ^26^ ^]^ The unique properties of wide bandgap (1.8–2.0 eV), high work function (>5.0 eV), p‐type conductivity (≈10^14^ cm^−3^), and high mobility (≈60 cm^2^ V^−1^ s^−1^) make t‐Se a promising p‐type interfacial layer for constructing efficient Sb_2_Se_3_ solar cells.^[^ ^26^, ^27^ ^]^ We developed a seed layer assisted successive CSS process to fabricate high‐crystalline Sb_2_Se_3_ films. The chemical and electrical properties of the Sb_2_Se_3_ surface were carefully analyzed. To reduce surface Se deficiency and the related downward band bending, reactive Se was introduced to compensate the surface Se deficiency and form an oriented 1D trigonal Se interlayer on the Sb_2_Se_3_ surface. It is found that the prepared t‐Se, which has (101) preferred orientation matches well with the crystal orientation of the Sb_2_Se_3_ absorber. Further, the p‐type t‐Se functionally worked as surface passivation and p‐type hole transport layer, facilitating the formation of n–i–p structured Sb_2_Se_3_ solar cells. As a consequence, an efficiency of 7.45% was achieved, which is the highest efficiency ever reported for superstrate Sb_2_Se_3_ solar cells prepared by CSS.

## 2 Results and Discussion

### 2.1 Fabrication of Sb_2_Se_3_ Films via Seed Layer Assisted Successive CSS Process

**Figure 1:**
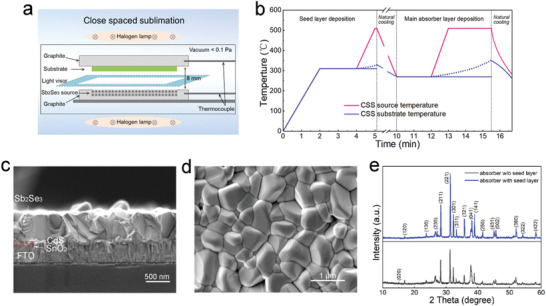
Fabrication and characterization of Sb_2_Se_3_ layer. a) Schematic diagram of the close spaced sublimation (CSS) apparatus for Sb_2_Se_3_ film deposition. b) The temperature profile for seed layer assisted successive CSS process. c) Cross‐sectional SEM image and d) top‐view SEM image of Sb_2_Se_3_ film. e) XRD spectra of the Sb_2_Se_3_ films deposited by seed layer assisted successive CSS process and single CSS process.

In this study, Sb_2_Se_3_ films were prepared by the CSS method according to our previous work with some modifications.^[^ ^19^ ^]^ A ceramic light visor was inserted between source and substrate to prevent overheating the graphite substrate holder by interactive light radiation (**Figure** **1**a). This optimization promoted more independent control of substrate and source temperatures. The maximum steady‐state temperature difference between the source and substrate was enlarged from ≈120 to ≈150 °C (the source temperature was 510 °C).

We developed a seed layer assisted successive CSS process to enhance preferred orientation and grain growth. As shown in Figure 1b, a thin Sb_2_Se_3_ seed layer (≈10 nm) was first deposited onto the CdS surface, then the main Sb_2_Se_3_ absorber layer (≈500 nm) was deposited on the seed layer. The two deposition processes were conducted in a successive manner without breaking the vacuum while keeping the substrate temperature higher than 270 °C. This successive high‐temperature process without low‐temperature stage can eliminate repeated nucleation on the grain surface and favor continuous grain growth.^[^ ^28^, ^29^ ^]^ Figure 1c,d shows the cross‐sectional and top‐view scanning electron microscopy (SEM) images of the Sb_2_Se_3_ film. It can be seen that the Sb_2_Se_3_ film has an excellent crystallinity with Sb_2_Se_3_ grains as tall as the thickness of the film (through‐thickness grains). The grains are densely packed and have a lateral size of 0.5–1 µm, which is about two times larger than that of the grains in films made by the conventional single‐step CSS process.^[^ ^19^ ^]^ The XRD patterns of the Sb_2_Se_3_ films deposited by seed layer assisted successive CSS process and single‐step CSS process are shown in Figure 1e. The two films had the same thickness and were measured under identical conditions. The XRD peak intensity of the Sb_2_Se_3_ film prepared by seed layer assisted successive CSS is much stronger than that of the film by single‐step CSS, indicating that seed layer assisted successive CSS process could strongly enhances the crystallinity. Furthermore, the film by seed layer assisted successive CSS shows a more (221) preferred orientation. As Sb_2_Se_3_ is composed of 1D (Sb_4_Se_6_)*_n_* ribbons stacked together via weak van der Waals interaction, strong (221) preferred orientation growth with through‐thickness grains is highly favored for efficient carrier transport and benign grain boundaries.^[^ ^2^ ^]^

### 2.2 Surface Se Deficiency and Band Bending

**Figure 2:**
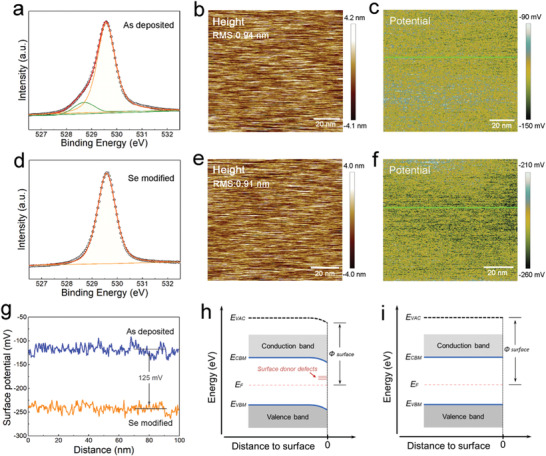
Surface chemical and electrical analysis. a) XPS peak of Sb 3d_5/2_ obtained from the surface of as‐deposited Sb_2_Se_3_. b) AFM image of the as‐deposited Sb_2_Se_3_ surface, and c) is the corresponding KPFM image obtained by scanning the same area. d) XPS peak of Sb 3d_5/2_ obtained from the surface of Se‐modified Sb_2_Se_3_. e) The AFM image of the Se‐modified Sb_2_Se_3_ surface, and f) the corresponding KPFM image obtained by scanning the same area. g) Potential profiles obtained along the two lines indicated in (c) and (f). Schematic diagrams of surface band bending of h) an as‐deposited Sb_2_Se_3_ film and i) a Se‐modified Sb_2_Se_3_ film.

In addition to structural properties, the chemical composition was characterized by high‐resolution X‐ray photoelectron spectroscopy (XPS). **Figure** **2**a shows the Sb 3d spectrum of the as‐deposited Sb_2_Se_3_ film. It was observed that the shoulder peak with lower binding energy at 528.7 eV was identified, which is representative of the reduced Sb elemental state or Se‐deficient component at the Sb_2_Se_3_ surface.^[^ ^30^, ^31^ ^]^ It has been reported that Sb_2_Se_3_ is slightly decomposed during high‐temperature process, leading to the formation of Se‐deficient Sb_2_Se_3‐_ *_x_* because of the high vapor pressure of Se.^[^ ^32^, ^33^, ^34^ ^]^ We further conducted XPS depth‐profile measurements to investigate the chemical distribution of Sb_2_Se_3_ absorber. Figure S2 of the Supporting Information shows XPS scans of the Sb 3d at various depths. The intensity of the shoulder peak decreased and disappeared with increasing depth, indicating that the detectable Se deficiency only exists within several nanometers from the surface. Because of the high vapor pressure of Se, the high‐temperature deposited Sb_2_Se_3_ film is prone to deviate from the stoichiometric ratio.^[^ ^18^, ^32^, ^33^, ^34^ ^]^ Theoretically, Se‐related defects produce a series of intrinsic doping levels in the bandgap and make Sb_2_Se_3_ p‐type under the Se‐rich condition and n‐type under the Se‐poor condition.^[^ ^18^, ^32^ ^]^ We assume that the surface Se deficiency is related to the change of chemical environment during the whole CSS process. When the source temperature is high (510 °C), due to the higher vapor pressure of Se, source vapor provides a Se‐rich environment around the substrate, leading to a less Se‐deficient condition. A sufficiently high Se content is critical to guarantee intrinsic p‐type conductivity in Sb_2_Se_3_ films. During the cooling process, however, the Se vapor around the hot substrate tends to diminish with decreasing source temperature. Under such circumstances, the deposited Sb_2_Se_3_ film may decompose or re‐evaporate, which lead to surface Se deficiency because of the high vapor pressure of Se.

In order to tackle the Se deficiency at the rear surface of Sb_2_Se_3_, we deposited an ultrathin Se layer (2 nm, monitored by crystal oscillator) on the Sb_2_Se_3_ surface by high‐vacuum thermal evaporation. The reactivity of Se on the Sb_2_Se_3_ surface was regulated by altering the substrate temperature. It is found that, when the substrate temperature was lower than 80 °C, the device performance changes were negligible. When the substrate temperature was elevated to 100 °C, enhanced spectral response at long wavelengths and enhanced device performance could be observed (Figure S3, Supporting Information). However, when the substrate temperature was higher than 110 °C, it was difficult to deposit Se on the Sb_2_Se_3_ surface. To understand the mechanism of the performance enhancement, the Se‐modified Sb_2_Se_3_ surface was characterized. In order to characterize the change in chemical composition with respect to the pure Sb_2_Se_3_ surface, the samples with Se coating were cleaned to make sure that no superfluous Se residue was present on the surface (Figure S4, Supporting Information). As shown in Figure 2d, the shoulder peak at 528.7 eV disappeared, and the Sb 3d spectrum shows only one symmetric peak at 529.6 eV corresponding to Sb^3+^ in Sb_2_Se_3_.^[^ ^31^, ^35^ ^]^ These observations provide evidence that the Se modification process is fairly effective in compensating surface Se deficiency of the Sb_2_Se_3_ absorber.

To further characterize the surface electrical properties of Sb_2_Se_3_ films, Kelvin probe force microscopy (KPFM) was employed to map the surface morphology and potential of the as‐deposited Sb_2_Se_3_ surface and the Se‐modified Sb_2_Se_3_ surface. To calibrate the potential value of the probe, an Au sample was measured together with the Sb_2_Se_3_ samples (Figure S5, Supporting Information). The atomic force microscopy (AFM) images (Figure 2b,e) demonstrate similar height morphology with root‐mean‐square (RMS) roughness in the range of 0.91–0.94 nm. Figure 2c,f,g shows the KPFM images and the corresponding line profiles of the surface potentials. It is observed that the average surface potential of the Sb_2_Se_3_ film decreased by 125 mV after Se modification. The surface potential difference relates quantitatively to the difference in surface work functions (*Ф*).^[^ ^36^, ^37^ ^]^ The surface potential measurements indicate a work function increase of 125 mV for the Se‐modified Sb_2_Se_3_ surface as compared to the as‐deposited Sb_2_Se_3_ surface. This is a considerable change for a 1.2 eV bandgap semiconductor. The reason for the work function variation of the Sb_2_Se_3_ surface with different chemical composition can be explained by means of the schematic band diagram in Figure 2h,i. In general, the work function of a semiconductor can be written as *Φ* = *E* ^a^ + Δ*E*_fn_, where *E* ^a^ is the electron affinity and Δ*E*_fn_ is the Fermi level position referenced to the bottom of the conduction band.^[^ ^36^ ^]^ In case of the as‐deposited Sb_2_Se_3_ surface, the Se‐deficiency related defects, such as Se vacancies, usually produce deep‐donor levels in the bandgap which may act as carrier recombination centers.^[^ ^18^, ^32^ ^]^ The deep‐donor states near the conduction band uplift the Fermi energy, resulting in a downward band bending, smaller Δ*E*_fn_, and decreased *Ф* at the surface (Figure 2h).^[^ ^36^, ^37^, ^38^ ^]^ For the Se‐modified Sb_2_Se_3_ surface, due to the fact that the Se deficiency was compensated, the position of the Fermi energy at the surface is consistent with that of bulk Sb_2_Se_3_, leading to a surface under more flat band conditions (Figure 2i).

It is worth pointing out that surface band bending or even band inversion has been found in several kinds of semiconductors.^[^ ^37^, ^38^, ^39^, ^40^ ^]^ Experimental results have indicated that, in the region of p–n junction interface, an inverted surface band bending in the absorber layer is beneficial for device performance due to the reduction of the recombination rate by shifting the electrical junction away from the defective physical junction interface.^[^ ^39^, ^40^ ^]^ However, at the back contact interface, an inverted surface band bending in the absorber layer is detrimental to the device performance.^[^ ^41^ ^]^ On the one hand, the downward valence band bending forms a hole barrier, which impedes holes transport to the back electrode, and on the other hand, due to the negative conduction band bending, a portion of the photogenerated electrons would diffuse backward and recombine with holes at the back surface. Hence, modification of surface Se‐deficiency is essential not only to passivate the Se‐deficiency related deep‐level defects but also to construct a favorable band structure for carrier transport at the surface/interface.

### 2.3 Interfacial Structure and Orientation

**Figure 3:**
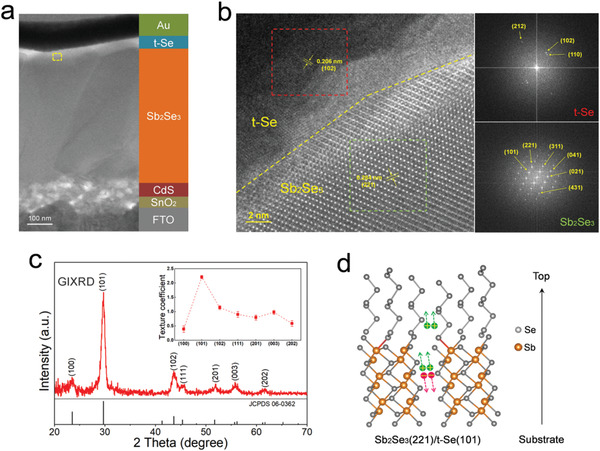
Interface structure and orientation. a) Cross‐sectional TEM image of the Sb_2_Se_3_ solar cell. b) HRTEM images of Sb_2_Se_3_/t‐Se interface and the Fourier transforms of corresponding lattice fringe. c) XRD pattern of t‐Se layer on the Sb_2_Se_3_ substrate using GIXRD mode. d) Atomistic model of [101]‐oriented 1D t‐Se on the (221) plane of Sb_2_Se_3_.

Considering the compatible chemical and electrical properties of Se with Sb_2_Se_3_, Se layers with different thicknesses were deposited as interfacial layer between Sb_2_Se_3_ and Au electrode. Figure S6 of the Supporting Information shows the device performance of the Sb_2_Se_3_ solar cells with Se layer thicknesses of 0, 10, 15, 20, and 30 nm. All devices with a Se layer modification show performance improvements. The solar cell with a 15 nm Se layer shows the highest power conversion efficiency with all device parameters improved. To analyze the interface structure, high‐resolution transmission electron microscopy (HRTEM) was conducted. The sample for cross‐sectional imaging was prepared by focused ion beam and a TEM image of the device is shown in **Figure** **3**a. Figure 3b shows the HRTEM image of Sb_2_Se_3_ interface taken from the dashed rectangle of Figure 3a. As expected, the Sb_2_Se_3_ displays an orthorhombic phase, judging from multiple interplane distances. On the other hand, the Se layer displays a trigonal phase with multiple typical interplane distances. The Fourier transforms of the corresponding lattice fringe of the orthorhombic Sb_2_Se_3_ and trigonal Se, and the major diffraction spots are indexed. Furthermore, grazing incidence X‐ray diffraction (GIXRD) with incidence angle of 0.5° was performed to confirm the crystal phases and lattice orientations of the Se layer on the Sb_2_Se_3_ surface (Figure 3c). All diffraction peaks are identified and indexed to the 1D trigonal Se (JCPDS 06–0362). The t‐Se layer shows preferential growth with an intensity of the (101) peak much higher than that of all other peaks. The orientation parameter of a crystalline plane (*hkl*) was quantitatively described by the texture coefficient (TC*_hkl_*).^[^ ^42^ ^]^ As can be seen in the inset of Figure 3c, TC(101) has a large value of 2.2, and all the other six TC values are close to or smaller than 1, indicating a strong (101) preferred orientation growth. Due to the distinct Q1D crystal characteristics, the disordered orientations at the Sb_2_Se_3_/t‐Se interface easily introduce trap centers.^[^ ^1^, ^2^, ^8^ ^]^ Especially for 1D t‐Se, it has been shown that the conductivity of t‐Se crystals is ≈10 times higher when measured parallel to the Se chains than in the perpendicular direction.^[^ ^43^ ^]^ The (101) preferred orientation of t‐Se indicates that the Se chains are perpendicular to the substrate and almost parallel to the Sb_2_Se_3_ ribbons with (221) preferred orientation (Figure 3d).^[^ ^44^ ^]^ The ordered and matched orientations at the Sb_2_Se_3_/t‐Se interface can reduce the possible interfacial defects and form a low‐resistance path for efficient carrier transport.

### 2.4 Interface Band Alignment

**Figure 4:**
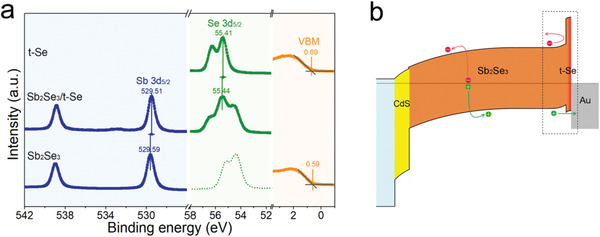
Band alignment at the Sb_2_Se_3_/t‐Se interface. a) The XPS spectra of Sb 3d, Se 3d and valence band in the bulk t‐Se, Sb_2_Se_3_/t‐Se interface, and bulk Sb_2_Se_3_. b) Schematic energy band diagram of the Sb_2_Se_3_ device drawn by XPS measurements.

To provide insight into the impact of t‐Se layer on the interfacial band structure, XPS measurements including core‐level spectroscopy and valence band (VB) spectroscopy were conducted at the Sb_2_Se_3_/t‐Se interface. The quantitative valence band offset (VBO) can be obtained by $\mathrm{VBO}\;=E_{\mathrm{VB}}^{b}\;-E_{\mathrm{VB}}^{a}+V_{\mathrm{bb}}$, where $E_{\mathrm{VB}}^{b}$ and $E_{\mathrm{VB}}^{a}$ are the energy positions of the valence band edges of bulk Sb_2_Se_3_ and bulk t‐Se, respectively, and *V*_bb_ is the band bending at the interface.^[^ ^45^ ^]^ The *V*_bb_ can be estimated from the core level energy shift between the bulk area and the interface, and can be calculated by $V_{\mathrm{bb}}=\left(E_{\mathrm{cl}}^{a}-E_{\mathrm{cl}}^{a}\left(i\right)\right)\;-\left(E_{\mathrm{cl}}^{b}\left(i\right)-E_{\mathrm{cl}}^{b}\right)$, where $E_{\mathrm{cl}}^{b}$ and $E_{\mathrm{cl}}^{a}$ are the core level energies of Sb 3d_5/2_ and Se 3d_5/2_ in the bulk Sb_2_Se_3_ and bulk t‐Se, respectively, and $E_{\mathrm{cl}}^{b}\left(i\right)$ and $E_{\mathrm{cl}}^{a}\left(i\right)$ are the core level energies of Sb 3d_5/2_ and Se 3d_5/2_ at the Sb_2_Se_3_/t‐Se interface, respectively. Note that the values in the equation are calculated under the assumption that binding energy below Fermi edge is negative. The XPS curves and the binding energies are shown in **Figure** **4**a. Considering that the Se element is included in both Sb_2_Se_3_ and t‐Se, we chose higher peak position (≈55.4 eV) as the core level energy of Se 3d_5/2_, which corresponds to the binding energy of Se 3d_5/2_ in t‐Se (Figure S4, Supporting Information). The difference of valence band maxima (VBM) of Sb_2_Se_3_ layer and t‐Se layer is 0.10 eV. The total *V*_bb_ and VBO at the Sb_2_Se_3_/t‐Se interface are 0.11 and 0.21 eV, respectively. To confirm the valence band alignment and band bending at the interface, ultraviolet photoelectric spectroscopy (UPS) was used to examine the energy levels (Figure S7, Supporting Information). The calculated VBM and work functions are 0.56 and 4.55 eV for the Sb_2_Se_3_ film, and 0.70 and 5.18 eV for the t‐Se film, respectively. The VBM values are in good agreement with those shown in Figure 4a. Besides, the higher work function of t‐Se favors an interface electrical field directed from Sb_2_Se_3_ to t‐Se, which corresponds to an upward band bending at the interface of Sb_2_Se_3_/t‐Se. Given that the bandgaps of bulk Sb_2_Se_3_ and t‐Se remain unchanged, the conduction band offset (CBO) can be calculated by $\mathrm{CBO}\;=(E_{\mathrm{gap}}^{b}\;-E_{\mathrm{gap}}^{a})+\mathrm{VBO}$. The bandgaps of the Sb_2_Se_3_ and t‐Se were measured to be 1.20 and 1.98 eV, respectively (Figure S8, Supporting Information), and the CBO was calculated to be −0.57 eV.

Figure 4b shows the band diagram of the solar cell with a layer stack of fluorine tin oxide (FTO)/CdS/Sb_2_Se_3_/t‐Se/Au. The Sb_2_Se_3_/t‐Se forms a favorable band alignment at the contact interface. The upward band bending at the Sb_2_Se_3_ surface along with the close VBM levels between the Sb_2_Se_3_ and t‐Se will facilitate the efficient extraction of holes from Sb_2_Se_3_ to electrode. At the same time, due to the higher conduction band maximum, the t‐Se layer acts as a back field reflector, which blocks photogenerated electrons from injecting into the back electrode and consequently reduces carrier recombination at the back contact. Furthermore, the p‐type conductivity (≈10^14^ cm^−3^, about one order of magnitude higher than Sb_2_Se_3_) and the preferred orientation of 1D t‐Se favors hole injection and transport in the t‐Se layer. Based on the discussions above, it can be concluded that the t‐Se layer functionally works as a p‐type hole transport material in the n–i–p device, by passivating the rear surface, facilitating both hole extraction and electron reflection. The matching interfacial properties enable to effectively suppress the interface recombination and reduce the series resistance of the devices, which is expected to deliver a significantly enhanced photovoltaic performance.

### 2.5 Device Performance and Analysis

**Figure 5:**
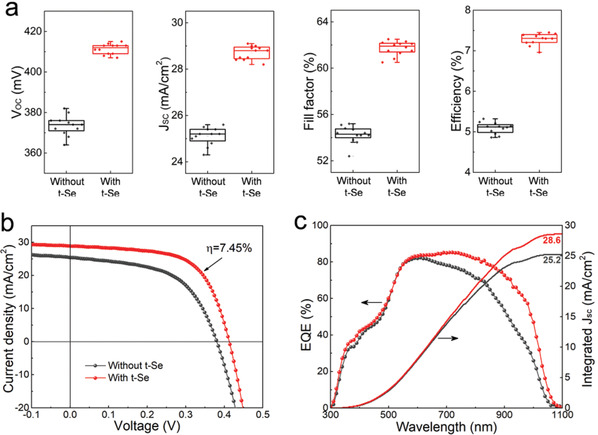
Device performance. a) Performance statistics of Sb_2_Se_3_ devices with and without t‐Se layer, 12 Sb_2_Se_3_ solar cells were fabricated per experimental condition. b) The *J*–*V* curves and c) EQE curves of the best performing Sb_2_Se_3_ devices with and without t‐Se layer.

As mentioned earlier, we examined the effect of t‐Se growth temperatures and layer thicknesses. Solar cells with 15 nm t‐Se layer deposited at 100 °C gave the best and most reproducible performance. **Figure** **5**a shows the statistic device performance of Sb_2_Se_3_ solar cells with and without 15 nm thick t‐Se layer. It is obvious that, compared to the control device, solar cells modified with t‐Se layers exhibit noticeably higher overall performance in terms of all photovoltaic parameters. The average power conversion efficiency (PCE) dramatically increases from ≈5.2% to over 7%. Figure 5b,c presents the current density–voltage (*J*–*V*) and external quantum efficiency (EQE) curves of the best devices with and without t‐Se measured under standard test conditions. The detailed device parameters are summarized in **Table** **1**. The parasitic resistances *R*_S_ and *R*_SH_ were extracted from the *J*–*V* curves using Site's method.^[^ ^46^ ^]^ Compared with the control devices, the open‐circuit voltage (*V*_OC_), short‐circuit current density (*J*_SC_), and fill factor (FF) of the device with t‐Se layer are enhanced by 8.7%, 13.8%, and 13.1%, respectively, resulting in a 40% improvement in PCE, from 5.32% to 7.45%. To the best of our knowledge, the obtained efficiency of 7.45% in this study is the highest efficiency value among all reported superstrate Sb_2_Se_3_ solar cells fabricated by CSS, and very close to the record efficiency (7.62%) of the superstrate Sb_2_Se_3_ solar cells, which is employed with toxic PbS hole transport layer.^[^ ^4^ ^]^ In addition, no hysteresis between forward and reverse scans was observed in the Sb_2_Se_3_ solar cell with t‐Se layer (Figure S9, Supporting Information). The EQE characterizations were carried out to verify the reliability of the *J*–*V* measurement. The integrated *J*_SC_ values from EQE curves are 25.2 and 28.6 mA cm^−2^ for Sb_2_Se_3_ solar cells without and with t‐Se layer, respectively, which are in good agreement with the values derived from the *J*–*V* measurements (Figure 5c).

**Table 1:** Photovoltaic performance parameters for the Sb_2_Se_3_ solar cells with and without t‐Se layer

Device	*J*_SC_ [mA cm^−2^]	*V*_OC_ [V]	FF [%]	PCE [%]	*R*_S_ [Ω cm^2^]	*R*_SH_ [Ω cm^2^]
w/o t‐Se	25.4	0.380	55.1	5.32	3.1	158
with t‐Se	28.9	0.413	62.3	7.45	2.2	249

**Figure 6:**
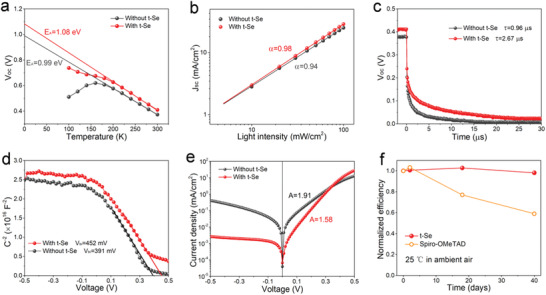
Device analysis. a) Temperature‐dependent *V*_OC_, and b) *J*_SC_ versus light intensity, and c) *V*_OC_ decay curves, and d) *C* ^−2^–*V* curves, and e) *J*–*V* curves under dark conditions of Sb_2_Se_3_ devices with and without t‐Se layer, respectively. f) Long‐term stability of Sb_2_Se_3_ devices with t‐Se layer and with Spiro‐OMeTAD layer.

In order to elucidate the origin of the performance improvement, specific device characterizations were performed. First, it is obviously seen from Figure 5c that the higher *J*_SC_ of solar cells with t‐Se layer is mainly results from spectral response enhancement in the long‐wavelength region (>600 nm). The EQE at the wavelength of 1000 nm is still about 50%, which is higher than most previously reported values (≈30%).^[^ ^16^, ^17^, ^20^, ^24^ ^]^ The EQE improvement in the long wavelength region (600–1100 nm) contributes to an integrated photocurrent increase of ≈3 mA cm^−2^. Due to the photons at the short‐wavelength region (<625 nm) have smaller absorption depth, the t‐Se layer does not absorb the photons passing through the Sb_2_Se_3_ absorber and contribute to the EQE enhancement (Figure S10, Supporting Information). It is known that the photons with long wavelength are generally absorbed at a deep position in the absorber layer, the increased EQE response at long wavelengths is mainly due to the sufficient generation and collection of carriers close to the deep or back side.^[^ ^46^ ^]^ Considering that the limited thickness of the Sb_2_Se_3_ layer (≈500 nm), the transport and recombination of carriers generated at deeply in the absorber layer can be easily affected by the rear surface or interface properties. With the introduction of t‐Se layer, the upward band bending in the surface region promotes holes drifting as well as injection via the back field. The passivated deep‐donor surface defects and the blocking effect of the t‐Se layer for electrons reduce the carrier recombination at the back interface. In addition, the depletion width (*x*_d_) of the main junction is found to be increased from 223 to 238 nm for the cell with the t‐Se layer, which strengthens the charge carrier separation and transport deeply in the Sb_2_Se_3_ absorber layer (Figure 6d). Overall, the favored band structure and suppressed recombination are responsible for the EQE enhancement at long wavelengths and the corresponding *J*_SC_ increase.

The carrier transport behavior was analyzed by temperature‐dependent *V*_OC_ and light intensity‐dependent *J*_SC_ measurements. As shown in **Figure** **6**a, the plot of *V*_OC_ versus temperature provides information about the activation energy (*E*_A_) of the main recombination mechanism.^[^ ^47^ ^]^ The *E*_A_ linearly extrapolated from the curve of *V*_OC_ versus temperature at 0 K shows a high value of 1.08 eV for the solar cells with a t‐Se layer, which is close to the bandgap of the Sb_2_Se_3_ absorber (1.20 eV), suggesting a dominant Schottky–Read–Hall recombination process in the space charge region. On the other hand, for the device without t‐Se, the *E*_A_ is calculated to be 0.99 eV, which is about 0.21 eV lower than the bandgap, indicating additional interface recombination and thus the *V*_OC_ is also limited as well.^[^ ^47^ ^]^ Figure 6b shows a linear dependence of the *J*_SC_ on the light intensity for both types of solar cells, in which both slopes of the lines are close to 1, indicating that both devices suffer negligible from space‐charge limited photocurrent. The relatively higher value of the slope of the device with t‐Se layer indicates that a better back contact leads to a much easier charge transport.^[^ ^16^ ^]^

The carrier recombination dynamics was quantitatively analyzed by transient *V*_OC_ decay measurements. In a typical solar cell under open‐circuit conditions, the decay time of *V*_OC_ upon removal of light is representative of the recombination time constant of the solar cell.^[^ ^5^, ^17^ ^]^ Figure 6c displays the *V*_OC_ decay curves. The calculated decay times are determined to be 2.67 and 0.96 µs for Sb_2_Se_3_ solar cells with and without t‐Se layer, respectively. The longer *V*_OC_ decay time and the better retardation of carrier recombination confirm the reduction of interface defects and the related electronic recombination loss for the device with t‐Se layer.

Mott–Schottky (*C* ^−2^–*V*) measurements were conducted to understand the origin of the *V*_OC_ improvement from the built‐in potential (*V*_bi_) variation. As shown in Figure 6d, compared to the device without t‐Se layer, the *V*_bi_ of the device with t‐Se layer increased from 0.391 to 0.452 V. As *V*_bi_ is positively correlated to *V*_OC_, the increased *V*_bi_ theoretically contributes to the improvement of *V*_OC_.^[^ ^17^ ^]^ Considering the identical CdS/Sb_2_Se_3_ junction structure and the same slopes of the linear regions, the increased *V*_bi_ can be attributed mainly to the band bending at the Sb_2_Se_3_/t‐Se interface. Further, the diode properties are determined from the corresponding dark *J*–*V* curves of the two cells, as shown in Figure 6e. The device with t‐Se layer demonstrates a better diode rectification, which is consistent with the increased shunt resistance (*R*_SH_) and the decreased series resistance (*R*_S_) (Table 1). The optimized parasitic resistances contribute to the higher FF. The reduced leakage current can be attributed to the reduced recombination current and the possible shunt paths. The smaller *R*_S_ of the t‐Se based device is due to the enhanced carrier extraction efficiency and the reduced Schottky barrier at the back interface (Figure S11, Supporting Information). The diode ideality factors (A) are calculated to be 1.58 and 1.91 for the cells with and without t‐Se layer. The variation in A between 1 and 2 depends on the energies of the deep defects that act as dominant trap states or the tunneling contribution.^[^ ^46^ ^]^ The lower value of A affirms the reduced trap‐assisted recombination in the device with t‐Se layer.

In addition, we studied the long‐term stability of the Sb_2_Se_3_ solar cell, and compared this to the cell with the commonly used organic Spiro‐OMeTAD HTL. The two devices have the same structure except the HTL materials. The details of the Sb_2_Se_3_ solar cell with Spiro‐OMeTAD HTL used in the present stability studies can be found in our previous work.^[^ ^48^ ^]^ Both devices were stored and measured in ambient environment without encapsulation. As shown in Figure 6f, the normalized efficiency of the Sb_2_Se_3_ solar cell with t‐Se retains ≈98% of its initial value after storage for 40 days. By contrast, the efficiency shows about 40% decline for the device with the Spiro‐OMeTAD HTL after the same storage duration. These results suggest that the chemically compatible t‐Se based all‐inorganic structure is an effective approach to fabricate highly efficient and stable Sb_2_Se_3_ solar cells.

## 3 Conclusion

In summary, we have demonstrated a compatible approach to modify the Sb_2_Se_3_ surface and construct efficient n–i–p Sb_2_Se_3_ solar cells. A seed layer assisted successive CSS process was developed to fabricate high‐crystalline Sb_2_Se_3_ film. The surface Se deficiency and negative surface band bending were discovered. Reactive Se was innovatively introduced to compensate surface Se deficiency and form an oriented 1D t‐Se interlayer on the Sb_2_Se_3_ surface. It is found that the t‐Se layer has a matched (101) preferred orientation, and functionally works as surface passivation and p‐type hole transport material. Benefiting from the suppressed recombination and enhanced band structure, the device performance was significantly improved. An efficiency as high as 7.45% was achieved, which is the highest efficiency reported for superstrate Sb_2_Se_3_ solar cells prepared by CSS. Besides, the all‐inorganic Sb_2_Se_3_ solar cell shows excellent device stability. This work provides a compatible component and device architecture design for highly efficient and stable Sb_2_Se_3_ solar cells.

## 4 Experimental Section

##### Solar Cell Fabrication

FTO‐coated glass with sheet resistance of 8 Ω sq^−1^ was cleaned by sonication with by acetone, ethanol, and deionized water for 10 min, respectively. A resistive 30 nm SnO_2_ buffer layer was deposited on the FTO surface by the RF magnetron sputtering technique at the substrate temperature of 200 °C. CdS layers with a thickness of ≈60 nm were prepared on glass/FTO/SnO_2_ substrates by chemical bath deposition at 85 °C from a solution composed of deionized water, cadmium acetate, ammonium acetate, and thiourea. The substrate with deposited CdS layer was then immersed into saturated CdCl_2_ methanol solution to dip‐coat a thin CdCl_2_ layer, and heat treated in a furnace in air atmosphere at a temperature of 380 °C for 10 min. The Sb_2_Se_3_ absorber layers were deposited by a seed layer assisted successive CSS process in a modified CSS facility, as sketched in Figure 1a,b. The source material is commercial Sb_2_Se_3_ powder with 99.999% purity. The deposition of Sb_2_Se_3_ layer includes two processes: seed layer deposition and main absorber layer deposition. The CSS process was conducted at a vacuum pressure of ≈0.1 Pa. There is no inert gas or reactive gas in the chamber. First, seed layer deposition: set source and substrate temperatures at 310 °C for 120 s, raise source temperature to 510 °C in 60 s, keep the substrate temperature at 310 °C and actually the temperature increases to ≈330 °C after 60 s, keep the source temperature at 510 °C for 10 s; then the source and substrate temperatures naturally cooling to 270 °C and begin the main absorber layer deposition: keep the source and substrate temperatures at 270 °C for 120 s, raise source temperature to 510 °C in 60 s, and then keep at 510 °C for 150 s, the substrate temperature is set at 270 °C the whole process and actually increases to ≈350 °C when the process is over, then turn off the power to stop the deposition and finally take the film out when it is cooled to 150 °C. After that, the Sb_2_Se_3_ film was rinsed with carbon disulfide (CS_2_, Aladdin, AR) for 1 min and then rinsed by deionized water. The Se films were deposited by thermal evaporation with 99.999% pure Se particles. The chamber was pumped to a pressure of less than 5 × 10^−4^ Pa before evaporation. The substrates were rear heated at an average rate of 10 °C min^−1^ from room temperature to the designed temperature. The deposition rate was monitored by crystal oscillator. The Deposition rate was kept at ≈2 Å s^−1^. Subsequently, gold back contacts (≈100 nm) were deposited by thermal evaporation in a vacuum chamber and the active area of individual device was defined as 0.126 cm^2^.

##### Sample Preparation

Samples for Sb_2_Se_3_ surface measurements: the as‐deposited Sb_2_Se_3_ after rinsed by CS_2_ solution for 1 min was defined as as‐deposited Sb_2_Se_3_; the Sb_2_Se_3_/t‐Se (2 nm) was first re‐evaporated in an RTP furnace for 60 s at 150 °C under a vacuum pressure below 0.1 Pa, then immersed in CS_2_ solution for 5 min and rinsed by deionized water, the obtained sample with pure Sb_2_Se_3_ surface without Se residue was defined as Se‐modified Sb_2_Se_3_. Samples for interface band alignment measurements: a 50 nm thick t‐Se film deposited on top of the Sb_2_Se_3_ substrate was defined as bulk t‐Se; a 3 nm thick t‐Se film on the Sb_2_Se_3_ substrate was defined as Sb_2_Se_3_/t‐Se interface; the Sb_2_Se_3_/t‐Se(3 nm) was re‐evaporated at 150 °C and immersed in CS_2_ solution, the sample with clean Sb_2_Se_3_ surface without Se residual was defined as bulk Sb_2_Se_3_. Sample t‐Se for GIXRD measurement: a 50 nm thick t‐Se film on the Sb_2_Se_3_ substrate.

##### Material Characterization

The film morphologies were characterized by SEM (FEI Apreo LoVac). The optical properties were measured by UV–vis spectrophotometer (Agilent Tech, Cary 5000). XRD and GIXRD: XRD measurements were performed by a Bruker D8 Advance X‐ray diffractometer with Cu K*α* as the radiation source. GIXRD pattern was performed with incidence angle of 0.5° and five consecutive measurements were collected and averaged into single spectra. TEM and HRTEM: Samples for TEM were prepared by ablating the device using a focused ion beam microscope. Pt layers were deposited on top of the device for protection. TEM and HRTEM images were taken on a JEOL JEM 3200FS TEM at 300 kV. AFM and KPFM: The AFM and KPFM measurements were performed on a Bruker Dimension Icon with Nanoscope V controller. All the KPFM images were collected in lift mode by using SCM–PIT probes with a spring constant of 2.8 N m^−1^ and a resonant frequency of 78.3 kHz. During all KPFM measurements, the optimal lift height is 30 nm. UPS and XPS: UPS and XPS characterizations were obtained with a Thermo Fisher Scientific K‐Alpha^+^, using the HeI (21.22 eV) emission line and Al K*α* radiation (1486.6 eV). X‐ray photoelectron depth profiling spectra were recorded by using Ar^+^ sputtering gun operated at 3 keV, rastering a 2.0 × 2.0 mm^2^ area with 5 s postetch delays.

##### Device Characterization

The *J*–*V* curves were measured using a Keithley 2400 source meter. The illumination was provided by a Newport Oriel 92192 solar simulator with an AM1.5G spectrum, operating at 100 mW cm^−2^, which was calibrated by a standard silicon solar cell (91150 V) from Newport. The EQE spectra were measured in AC mode on a spectrum corresponding system (Enlitech QE‐R), calibrated by Si reference solar cell. Capacitance–voltage (*C*–*V*) was measured with the bias voltage varying from −0.5 to 0.5 V at room temperature under near‐dark condition. Besides, the measurement frequency was set to 100 kHz and the AC modulation voltage was set to 20 mV. The voltage dependent depletion width was calculated according the formulation *x*_d_ = *εA*/*C*(*V*), where *x*_d_ is the depletion width, *ɛ* is the permittivity of Sb_2_Se_3_, *A* is the device area, and *C*(*V*) is voltage dependent capacitance, respectively. For low temperature *J*–*V* measurements, the devices were placed in a closed‐cycle nitrogen cryostat, and the temperature was varied from 100 to 300 K with a step size of 20 K. A LabVIEW control program was used to operate the temperature controller and the Keithley 2400 source measurement unit for current and voltage data acquisition. Light intensity *J*–*V* measurements and transient photovoltage *V*_OC_ decay curves were measured by an electrochemical workstation (ZAHNER CIMPS, Germany). The light source is the standard wideband white LED light source for ZAHNER CIMPS. The light intensity changed from 1000 to 100 W m^−2^ in light intensity *J*–*V* measurements. The light intensity used in transient photovoltage *V*_OC_ decay measurements was 1000 W m^−2^.

## Conflict of Interest

The authors declare no conflict of interest.

## Supporting information

Supporting Information
