# Supplementary material for: Efficient and Stable Planar n–i–p Sb2Se3 Solar Cells Enabled by Oriented 1D Trigonal Selenium Structures
Source: Adv Sci (Weinh). 2020 Jun 30;7(16):2001013. doi: 10.1002/advs.202001013 (PMC7435233; doi:10.1002/advs.202001013)
Supplement: Supplementary file 1 — Supporting Information [file ADVS-7-2001013-s001.pdf]

Copyright WILEY-VCH Verlag GmbH & Co. KGaA, 69469 Weinheim, Germany, 2020.

Supporting Information

Efficient and Stable Planar n-i-p Sb_2Se_3 Solar Cells Enabled by Oriented One-dimensional Trigonal Selenium Structures

Kai Shen, Yu Zhang, Xiaoqing Wang, Chizhu Ou, Fei Guo, Hongbing Zhu, Cong Liu, Yanyan Gao, Ruud E. I. Schropp, Zhiqiang Li, Xianhu Liu, Yaohua Mai**

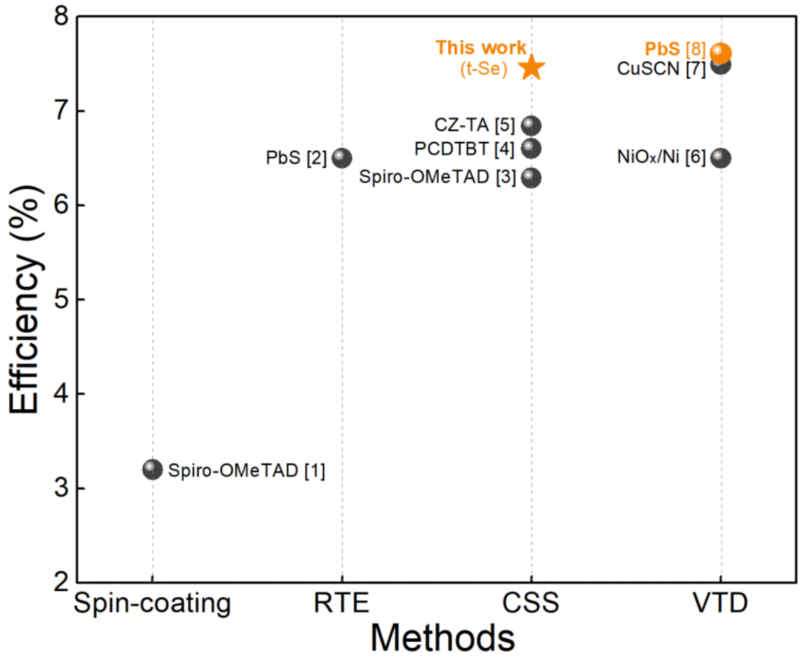

Figure S1 The efficiency versus Sb_2Se_3 deposition method for the preparation of superstrate Sb_2Se_3 solar cells with different contact/hole transport materials.[1-8] The efficiency (7.45%) obtained in this

work is the highest efficiency among all the reported superstrate Sb_2Se_3 solar cells fabricated by CSS, and very close to the record efficiency (7.62%) of the superstrate Sb_2Se_3 solar cell. It's noted that, the VTD-devices with higher efficiencies 7.62% and 7.50% used PbS and CuSCN as hole transport layer, respectively. The toxicity of Pb and high-mobility Cu impurity limit the safety and long-term stability of the devices.

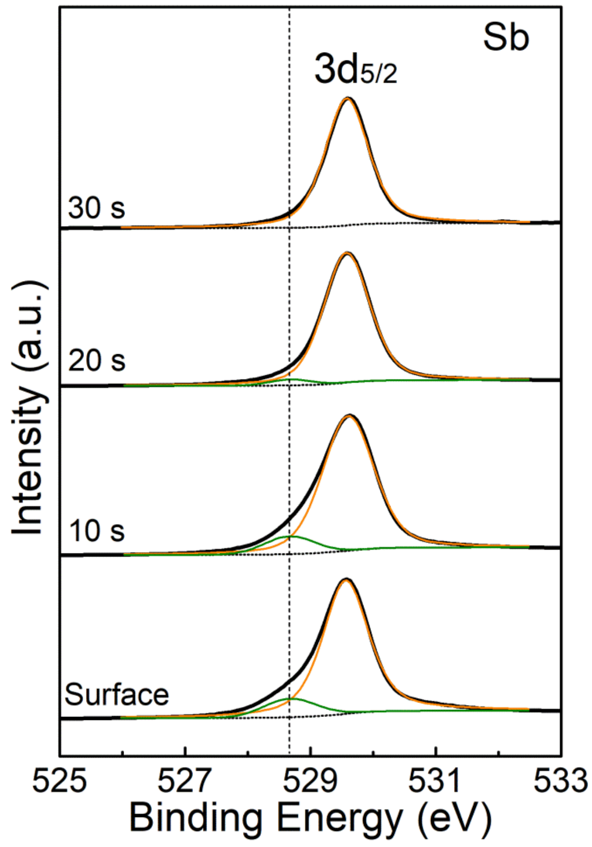

Figure S2 The Sb 3d peaks of Sb_2Se_3 surface versus the etching time. The etching rate is about 8 nm/min. The intensities of shoulder peaks at 528.7 eV decreased and disappeared with the increase of depth, which indicates that the detectable Se deficiency only exists within several nanometers of the surface.

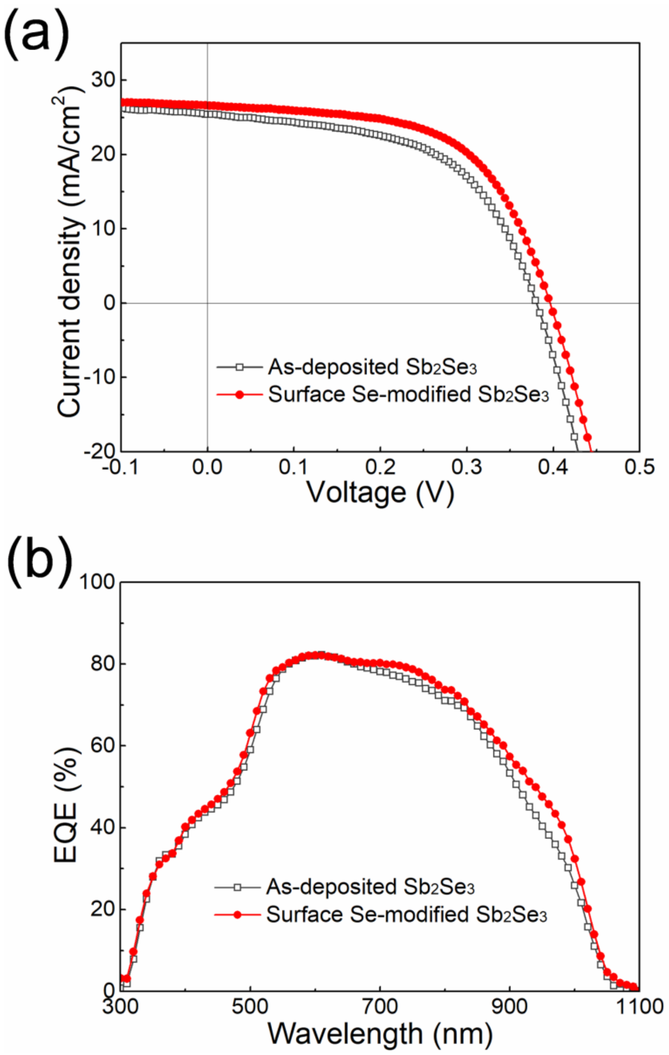

Figure S3 a) The J-V curves and b) EQE curves of Sb_2Se_3 solar cells with as-deposited Sb_2Se_3 and with surface Se-modified Sb_2Se_3. The device with Se-modified Sb_2Se_3 showed improved device performance and enhanced spectral response at the long wavelength.

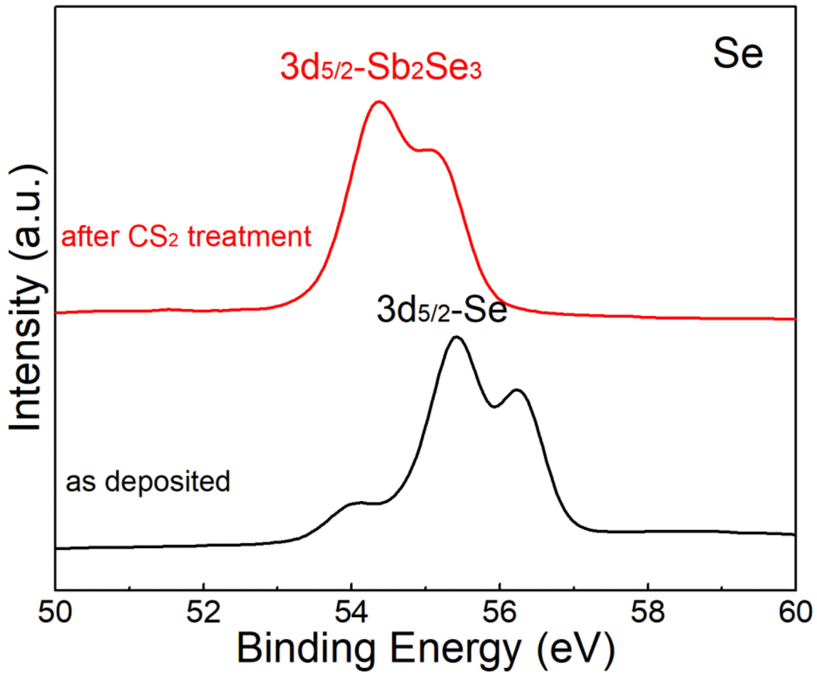

Figure S4 The Se $3d_{5/2}$ peaks of the surface of Sb_2Se_3/t-Se(2 nm) sample before and after CS_2 treatment. After re-evaporated at 150 ℃and immersed in CS_2 solution, the surface of the sample was pure Sb_2Se_3 surface without Se residue.

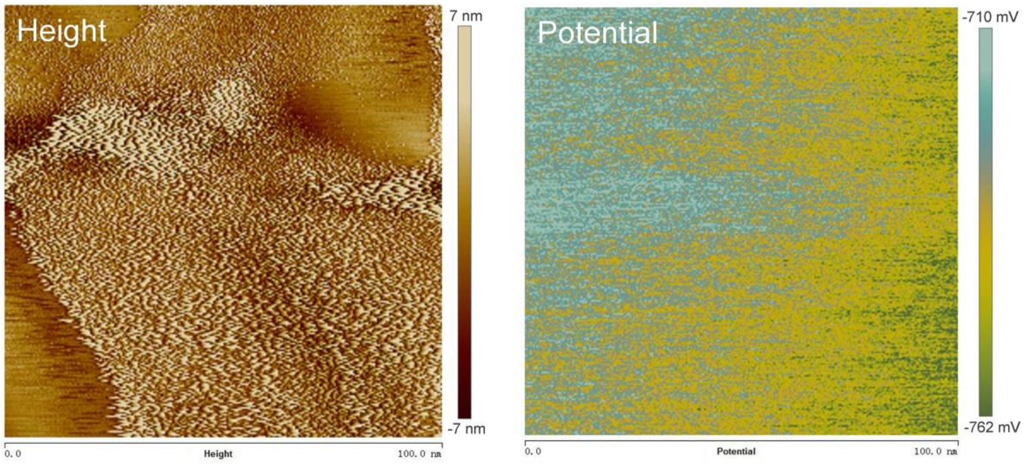

Figure S5 The AFM and KPFM images of Au film (~100 nm). The Au sample was measured together with the Sb_2Se_3 samples in Figure 2 to calibrate the potential value of the probe. The KPFM results indicate a work-function difference of ~500 mV between the Se modified Sb_2Se_3 surface and Au surface, which is consistent with the work-function value of Sb_2Se_3 calculated by UPS.

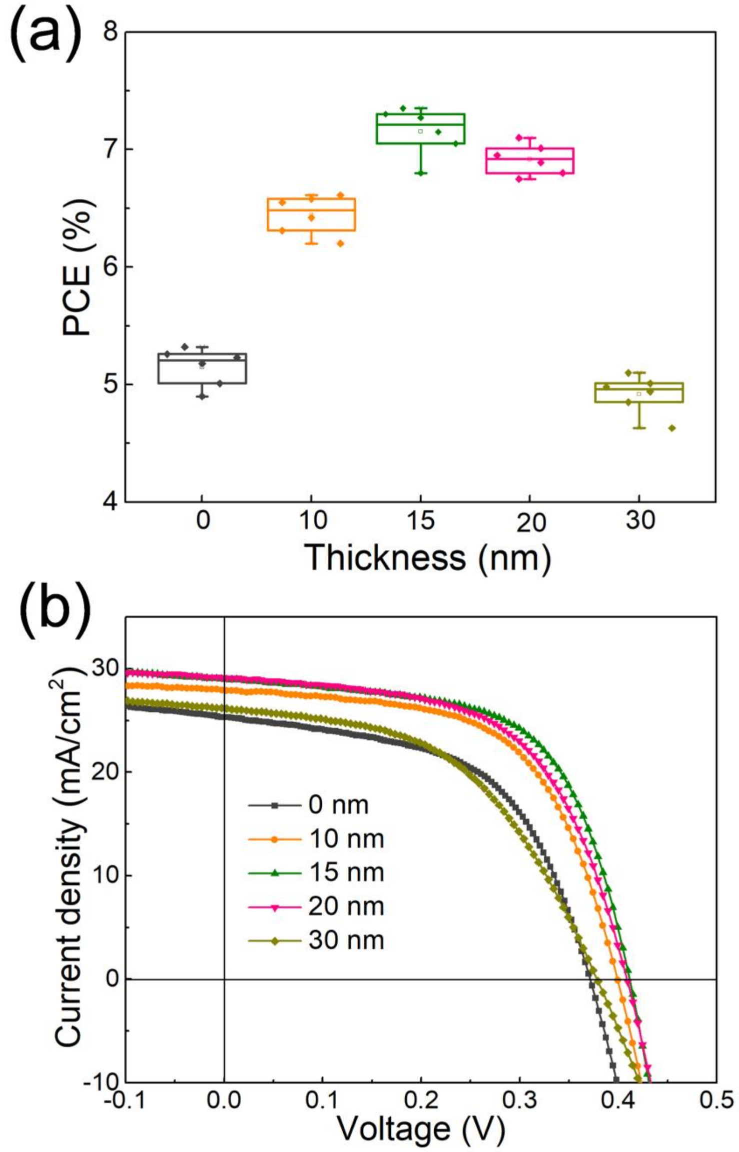

Figure S6 (a) The statistic PCEs and (b) the representative J-V curves of Sb_2Se_3 solar cells with different t-Se layer thickness. Most of the devices with t-Se layer presented performance improvements. Solar cells with 15 nm t-Se layer showed the highest power conversion efficiency.

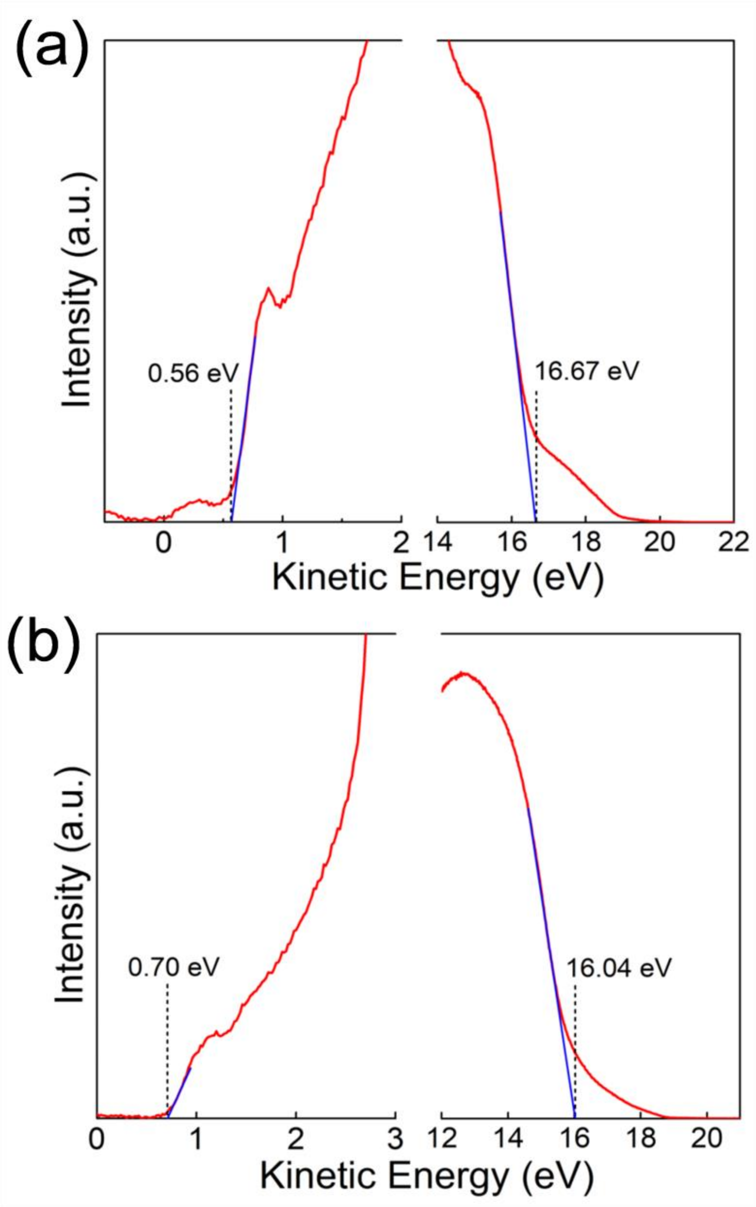

Figure S7 The UPS cutoff spectra of a) Sb_2Se_3 film and b) t-Se film. The measurement use the HeI (21.22 eV) emission line. The calculated VBM and work functions are 0.56 eV and 4.55 eV for Sb_2Se_3 film, and 0.70 eV and 5.18 eV for t-Se film, respectively.

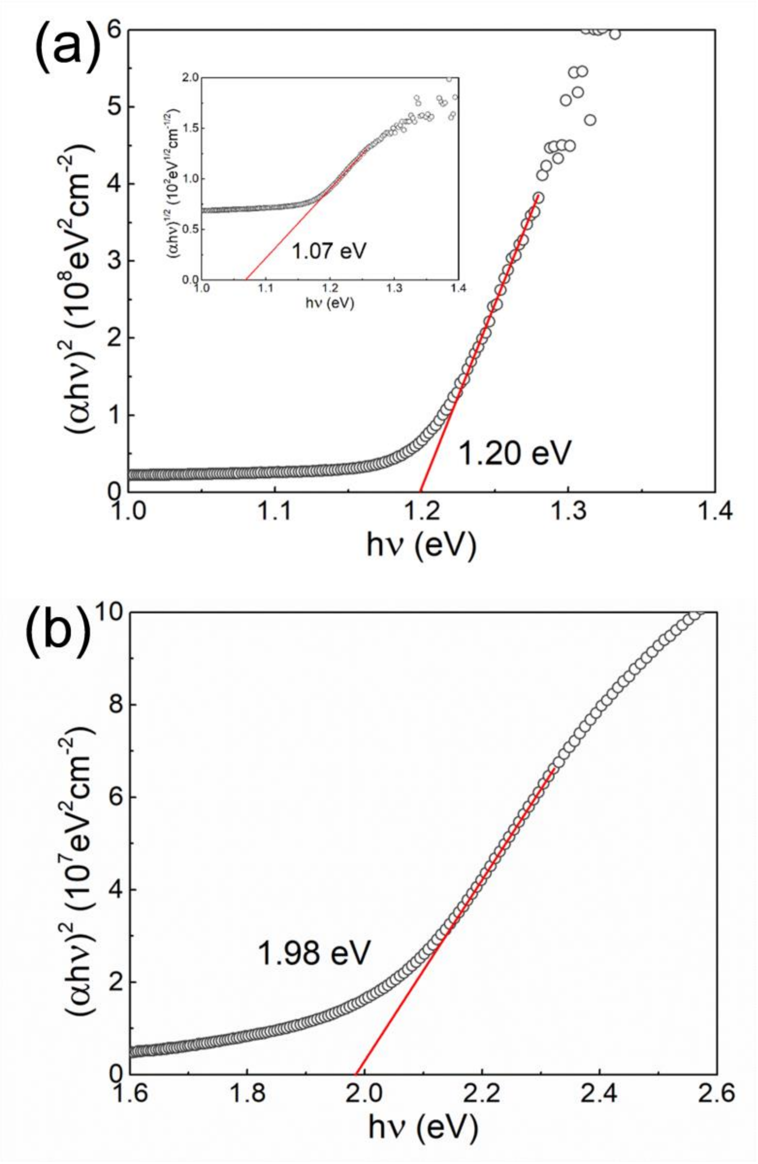

Figure S8 (a) Tauc plot (n = 2, direct, E_g = 1.20 eV; and insert, n = 1/2, indirect, E_g = 1.07 eV) for Sb_2Se_3 film. (b) Tauc plot for t-Se film, E_g = 1.98 eV.

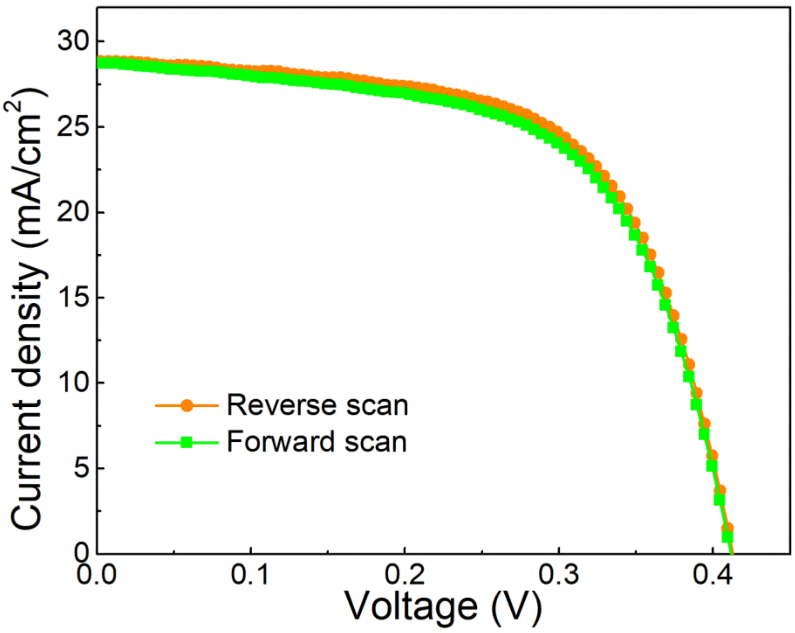

Figure S9 The *J-V* curves of the Sb_2Se_3 solar cell with t-Se layer measured in both forward and reverse scan directions. No hysteresis between forward and reverse scans was observed.

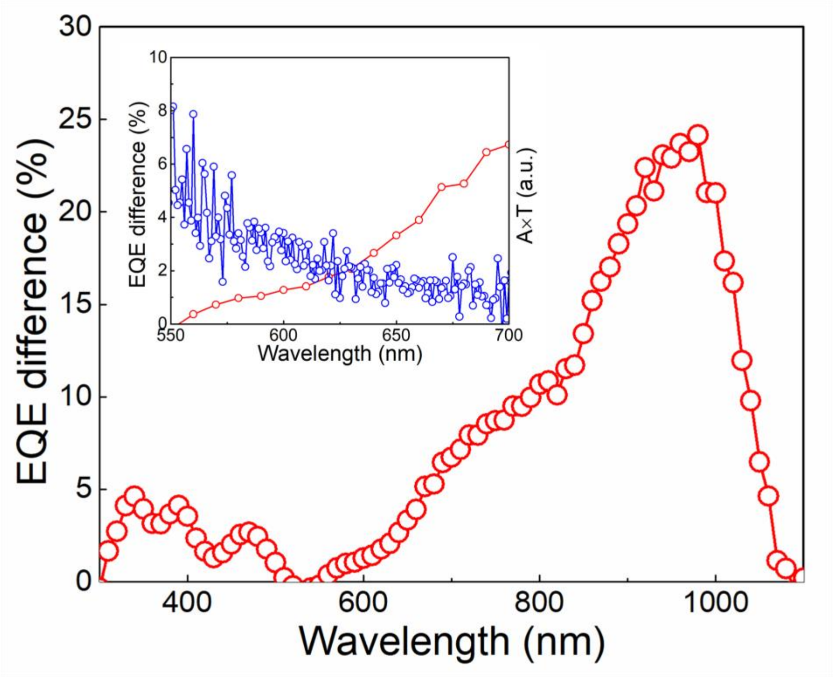

Figure S10 The difference between the EQE values of the Sb_2Se_3 solar cells with and without the t-Se layer. A is the absorption of t-Se and T is the transmittance of Sb_2Se_3 film. At the wavelength of ~625 nm (corresponding to the bandgap of t-Se: 1.98 eV), the total EQE enhancement is relatively small (<3%). Further, there are no obvious peaks both for EQE difference and light absorption by t-Se at 625 nm (see the inset), which indicates that the t-Se layer doesn’t absorb the photons passing through the Sb_2Se_3 absorber and contribute to the EQE enhancement.

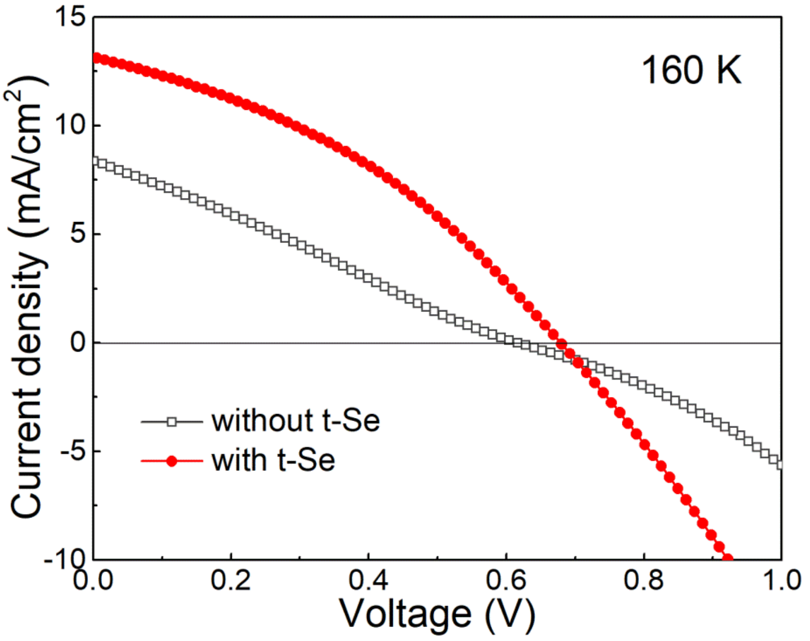

Figure S11 The J-V curves of Sb_2Se_3 solar cells with and without t-Se at 160 K. The device without t-Se appeared distinct rollover at high bias, while the device with t-Se behaved normal J-V curve. It suggested that t-Se structure reduced the Schottky barrier at the back contact.

References:

[1] X. Wang, R. Tang, Y. Yin, H. Ju, C. Zhu and T. Chen, *Solar Energy Materials and Solar Cells*, **2019**, 189, 5.

[2] C. Chen, L. Wang, L. Gao, D. Nam, D. Li, K. Li, Y. Zhao, C. Ge, H. Cheong, H. Liu, H. Song and J. Tang, *ACS Energy Letters*, **2017**, 2, 2125.

[3] C. Ou, K. Shen, Z. Li, H. Zhu, T. Huang and Y. Mai, *Solar Energy Materials and Solar Cells*, **2019**, 194, 47.

[4] O. S. Hutter, L. J. Phillips, K. Durose and J. D. Major, *Solar Energy Materials and Solar Cells*, **2018**, 188, 177.

[5] D.-B. Li, X. Yin, C. R. Grice, L. Guan, Z. Song, C. Wang, C. Chen, K. Li, A. J. Cimaroli, R. A. Awni, D. Zhao, H. Song, W. Tang, Y. Yan and J. Tang, *Nano Energy*, **2018**, 49, 346.

[6] J. Zhang, R. Kondrotas, S. Lu, C. Wang, C. Chen and J. Tang, *Solar Energy*, **2019**, 182, 96.

[7] K. Li, S. Wang, C. Chen, R. Kondrotas, M. Hu, S. Lu, C. Wang, W. Chen and J. Tang, *Journal of Materials Chemistry A*, **2019**, 7, 9665.

[8] K. Li, C. Chen, S. Lu, C. Wang, S. Wang, Y. Lu and J. Tang, *Advanced Materials*, **2019**, 31, 1903914.
